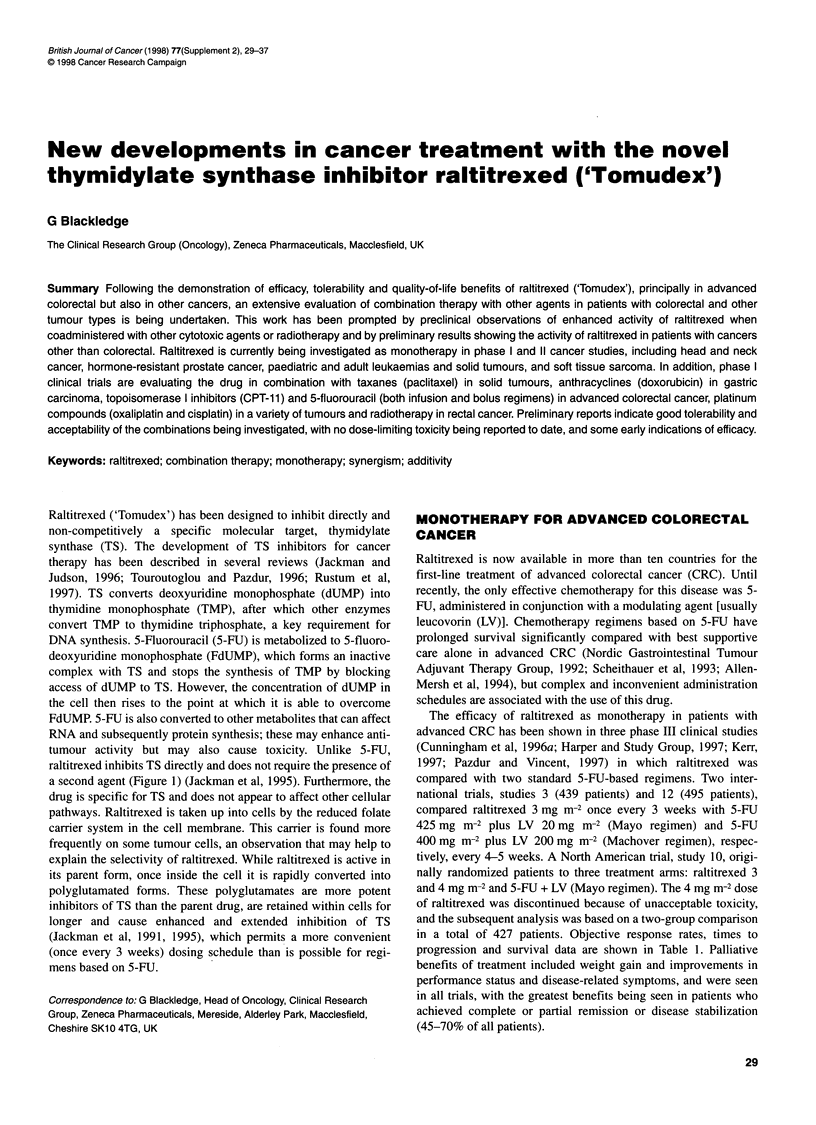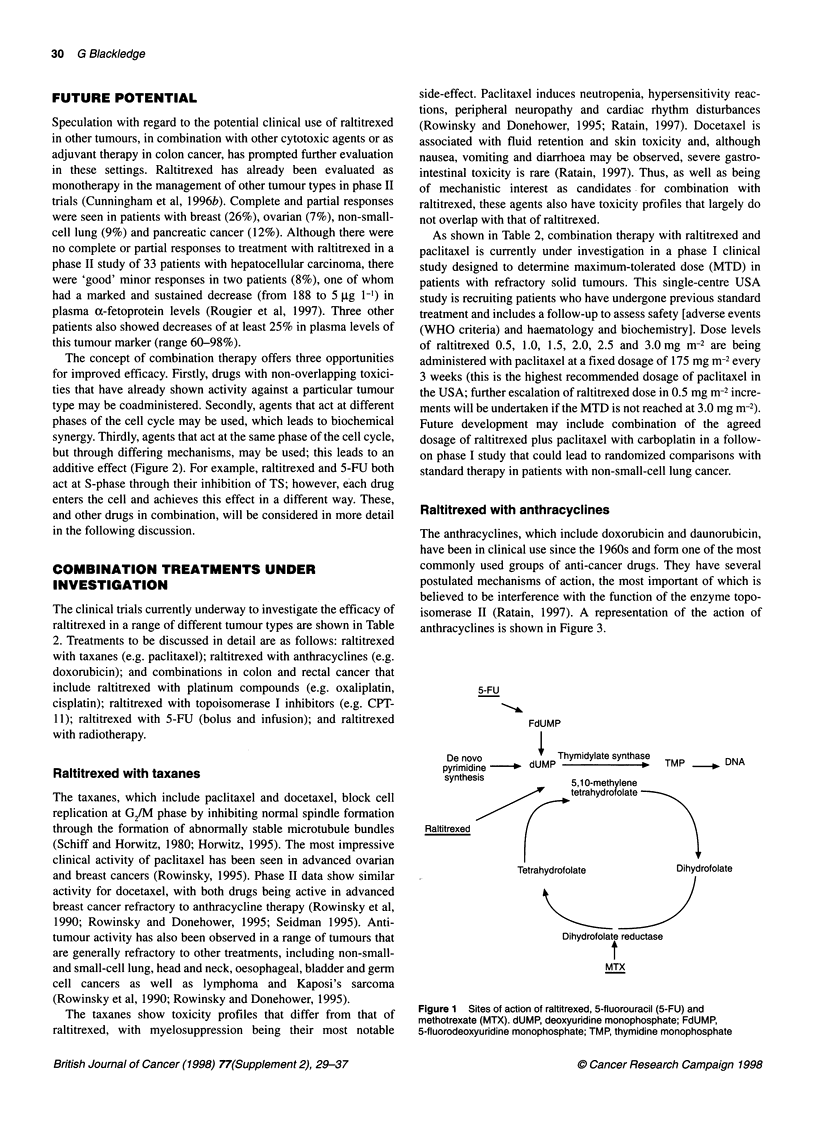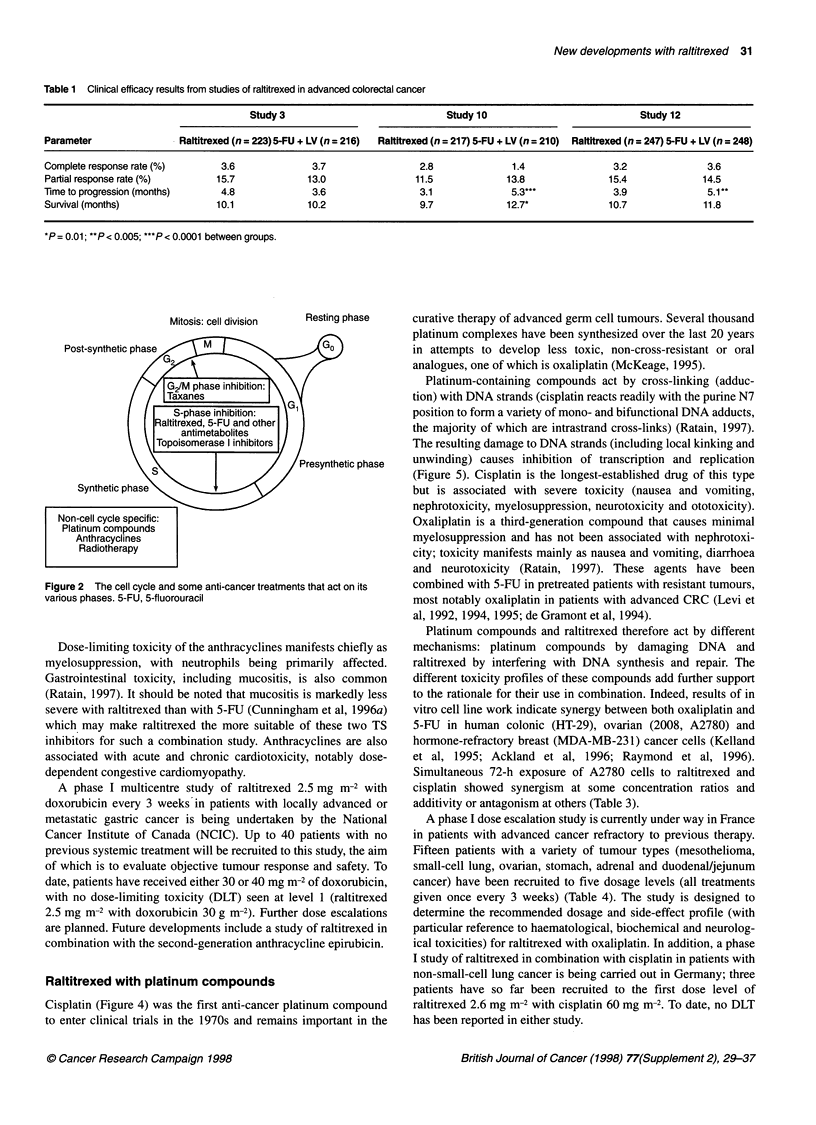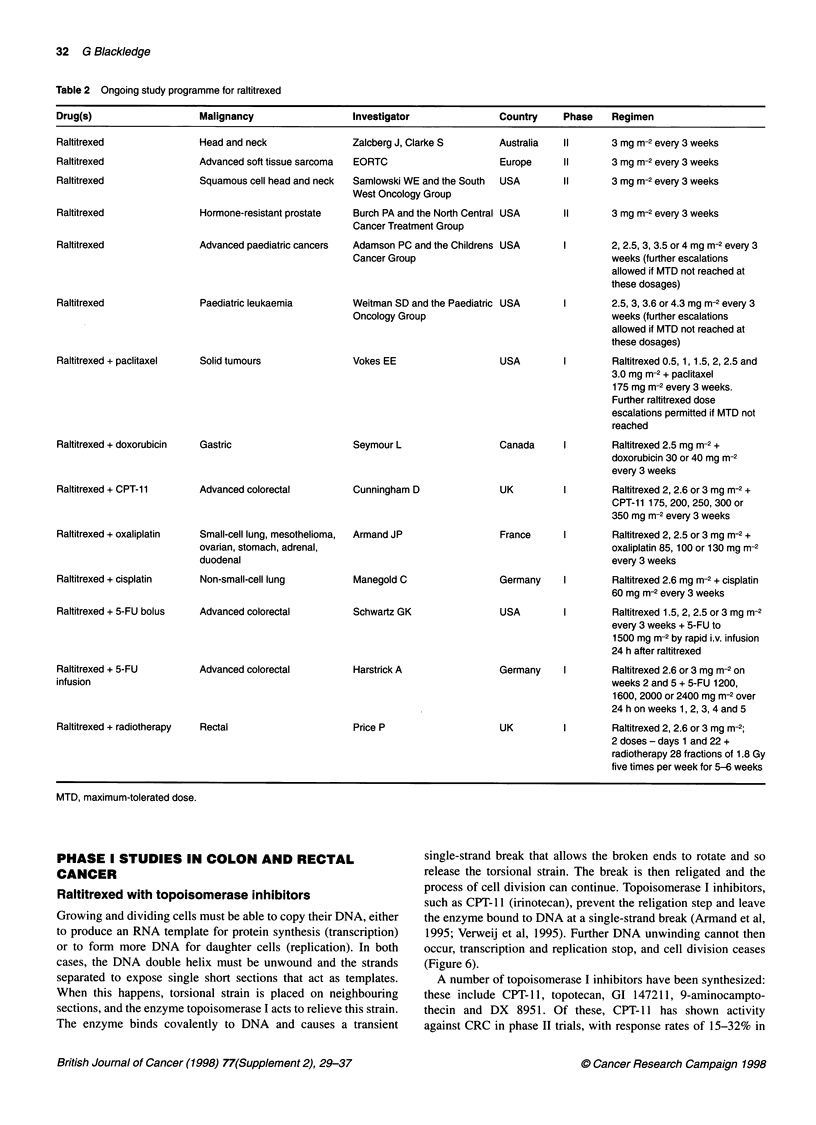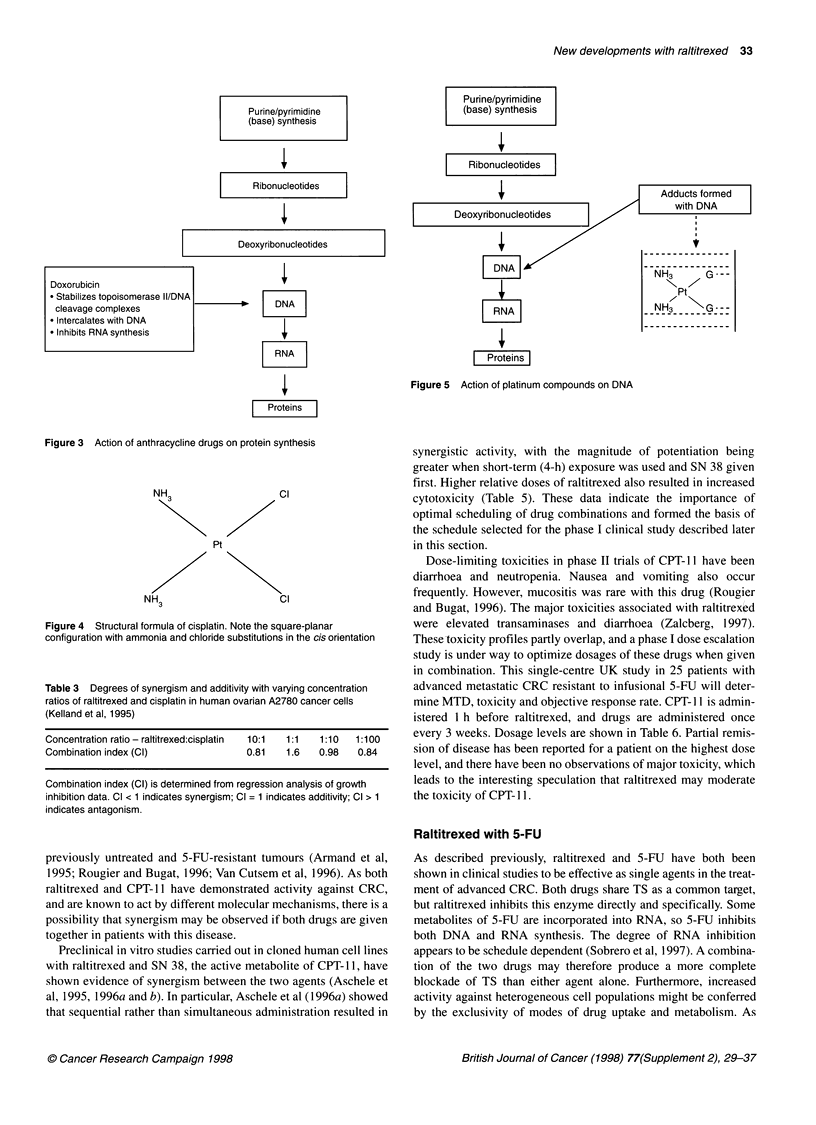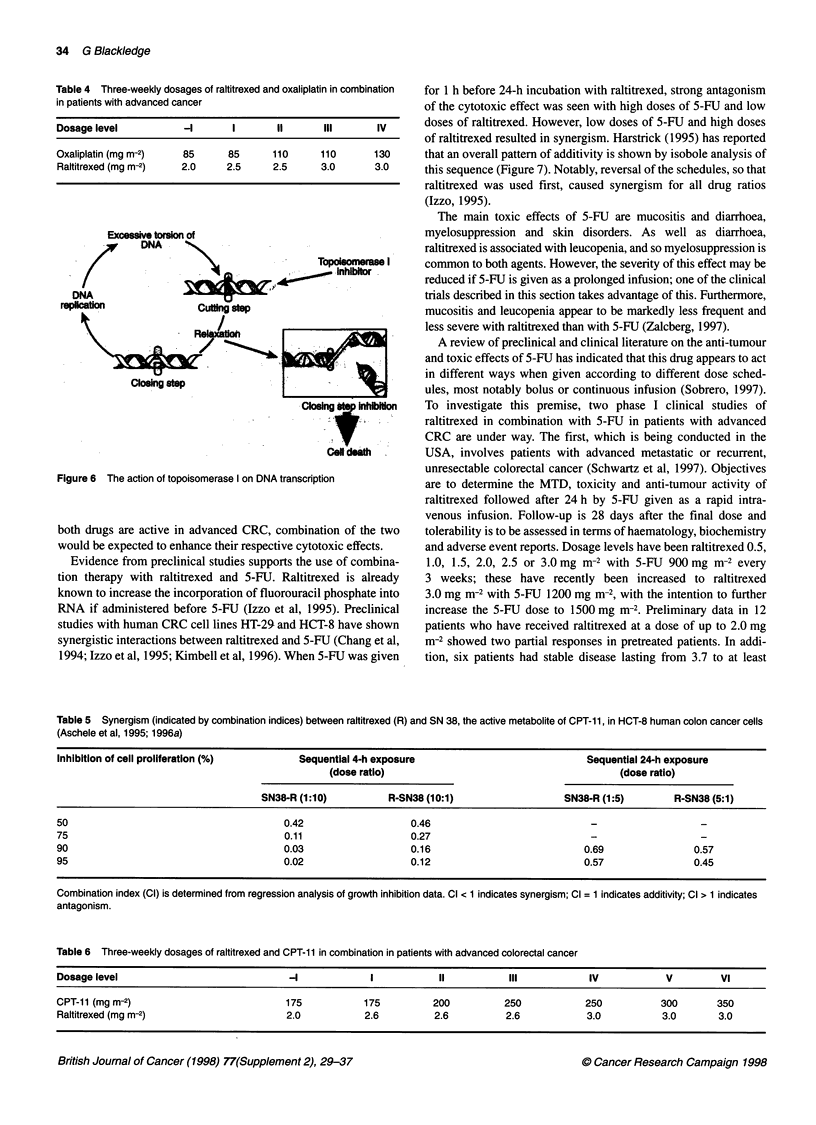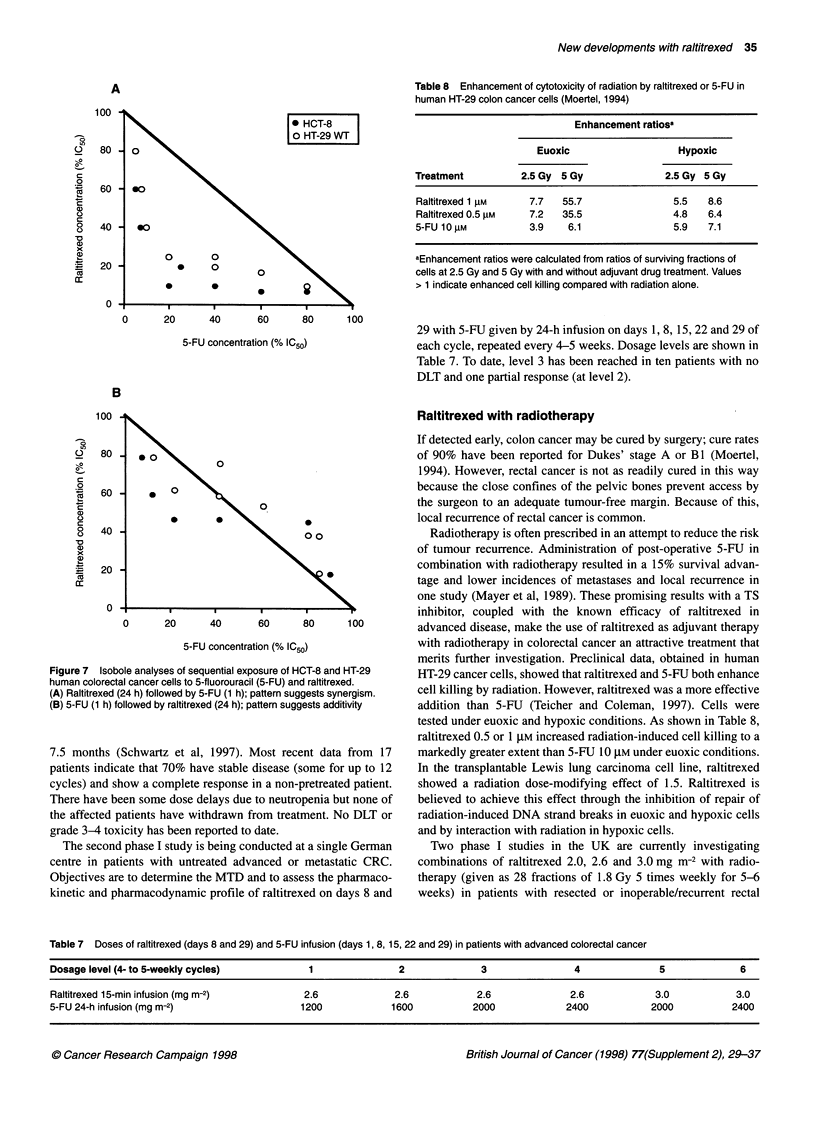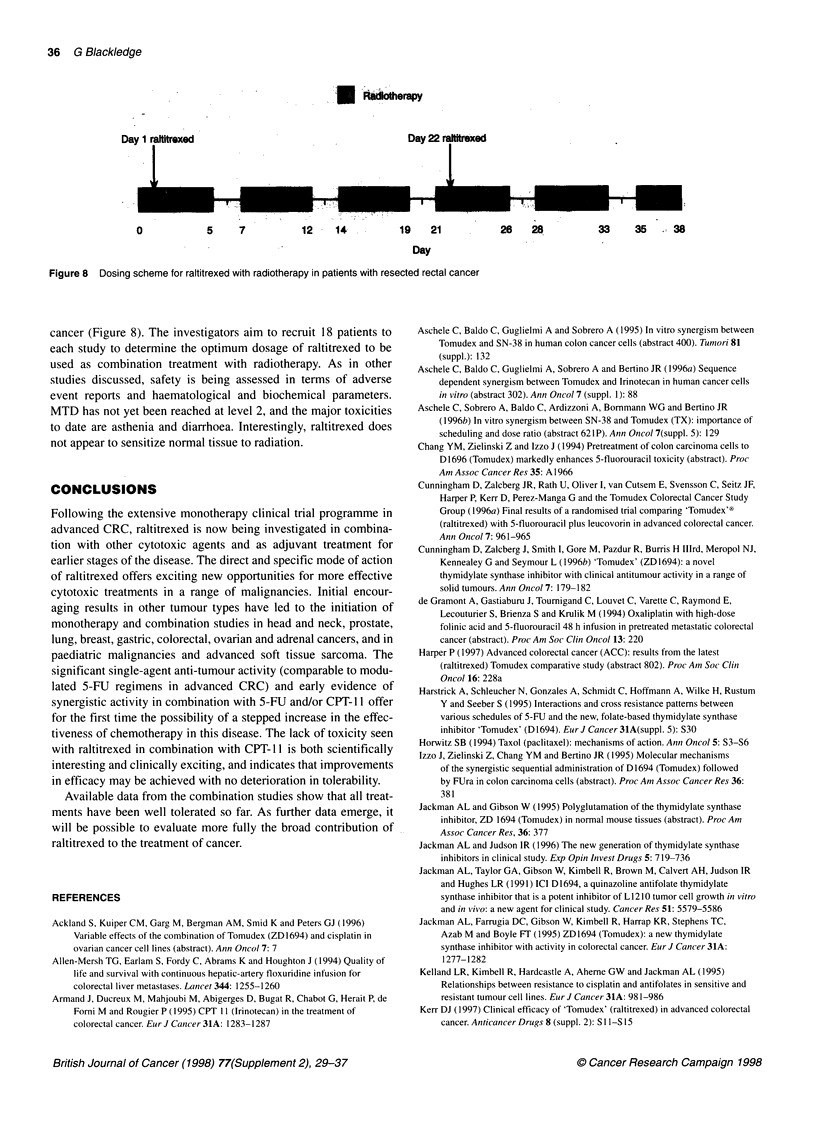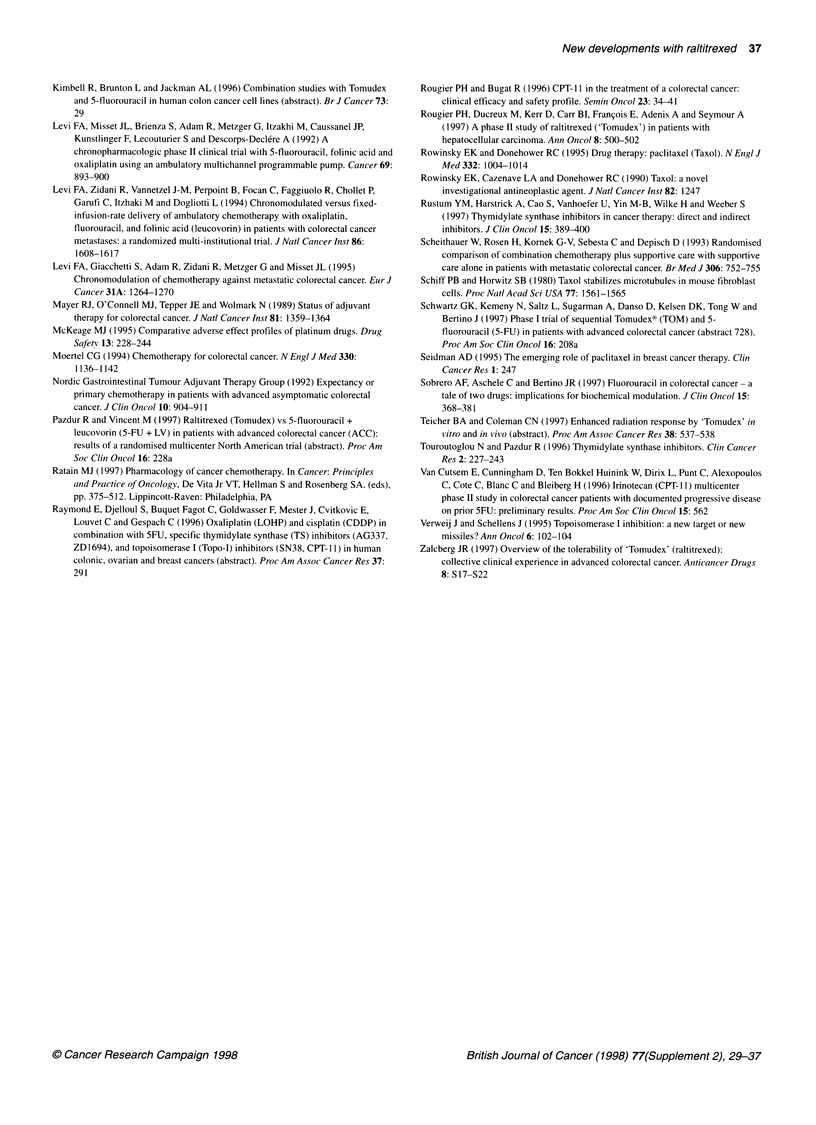# New developments in cancer treatment with the novel thymidylate synthase inhibitor raltitrexed ('Tomudex').

**DOI:** 10.1038/bjc.1998.423

**Published:** 1998

**Authors:** G. Blackledge

**Affiliations:** The Clinical Research Group (Oncology), Zeneca Pharmaceuticals, Macclesfield, Cheshire, UK.

## Abstract

Following the demonstration of efficacy, tolerability and quality-of-life benefits of raltitrexed ('Tomudex'), principally in advanced colorectal but also in other cancers, an extensive evaluation of combination therapy with other agents in patients with colorectal and other tumour types is being undertaken. This work has been prompted by preclinical observations of enhanced activity of raltitrexed when coadministered with other cytotoxic agents or radiotherapy and by preliminary results showing the activity of raltitrexed in patients with cancers other than colorectal. Raltitrexed is currently being investigated as monotherapy in phase I and II cancer studies, including head and neck cancer, hormone-resistant prostate cancer, paediatric and adult leukaemias and solid tumours, and soft tissue sarcoma. In addition, phase I clinical trials are evaluating the drug in combination with taxanes (paclitaxel) in solid tumours, anthracyclines (doxorubicin) in gastric carcinoma, topoisomerase I inhibitors (CPT-11) and 5-fluorouracil (both infusion and bolus regimens) in advanced colorectal cancer, platinum compounds (oxaliplatin and cisplatin) in a variety of tumours and radiotherapy in rectal cancer. Preliminary reports indicate good tolerability and acceptability of the combinations being investigated, with no dose-limiting toxicity being reported to date, and some early indications of efficacy.


*British Journal of Cancer* (1998) **77**(Supplement 2), 29–37
© 1998 Cancer Research Campaign

### New developments in cancer treatment with the novel thymidylate synthase inhibitor raltitrexed ('Tomudex')

**G Blackledge**

The Clinical Research Group (Oncology), Zeneca Pharmaceuticals, Macclesfield, UK

**Summary** Following the demonstration of efficacy, tolerability and quality-of-life benefits of raltitrexed ('Tomudex'), principally in advanced colorectal but also in other cancers, an extensive evaluation of combination therapy with other agents in patients with colorectal and other tumour types is being undertaken. This work has been prompted by preclinical observations of enhanced activity of raltitrexed when coadministered with other cytotoxic agents or radiotherapy and by preliminary results showing the activity of raltitrexed in patients with cancers other than colorectal. Raltitrexed is currently being investigated as monotherapy in phase I and II cancer studies, including head and neck cancer, hormone-resistant prostate cancer, paediatric and adult leukaemias and solid tumours, and soft tissue sarcoma. In addition, phase I clinical trials are evaluating the drug in combination with taxanes (paclitaxel) in solid tumours, anthracyclines (doxorubicin) in gastric carcinoma, topoisomerase I inhibitors (CPT-11) and 5-fluorouracil (both infusion and bolus regimens) in advanced colorectal cancer, platinum compounds (oxaliplatin and cisplatin) in a variety of tumours and radiotherapy in rectal cancer. Preliminary reports indicate good tolerability and acceptability of the combinations being investigated, with no dose-limiting toxicity being reported to date, and some early indications of efficacy.

**Keywords:** raltitrexed; combination therapy; monotherapy; synergism; additivity

Raltitrexed ('Tomudex') has been designed to inhibit directly and non-competitively a specific molecular target, thymidylate synthase (TS). The development of TS inhibitors for cancer therapy has been described in several reviews (Jackman and Judson, 1996; Touroutoglou and Pazdur, 1996; Rustum et al, 1997). TS converts deoxyuridine monophosphate (dUMP) into thymidine monophosphate (TMP), after which other enzymes convert TMP to thymidine triphosphate, a key requirement for DNA synthesis. 5-Fluorouracil (5-FU) is metabolized to 5-fluorodeoxyuridine monophosphate (FdUMP), which forms an inactive complex with TS and stops the synthesis of TMP by blocking access of dUMP to TS. However, the concentration of dUMP in the cell then rises to the point at which it is able to overcome FdUMP. 5-FU is also converted to other metabolites that can affect RNA and subsequently protein synthesis; these may enhance anti-tumour activity but may also cause toxicity. Unlike 5-FU, raltitrexed inhibits TS directly and does not require the presence of a second agent (Figure 1) (Jackman et al, 1995). Furthermore, the drug is specific for TS and does not appear to affect other cellular pathways. Raltitrexed is taken up into cells by the reduced folate carrier system in the cell membrane. This carrier is found more frequently on some tumour cells, an observation that may help to explain the selectivity of raltitrexed. While raltitrexed is active in its parent form, once inside the cell it is rapidly converted into polyglutamated forms. These polyglutamates are more potent inhibitors of TS than the parent drug, are retained within cells for longer and cause enhanced and extended inhibition of TS (Jackman et al, 1991, 1995), which permits a more convenient (once every 3 weeks) dosing schedule than is possible for regimens based on 5-FU.

*Correspondence to:* G Blackledge, Head of Oncology, Clinical Research Group, Zeneca Pharmaceuticals, Mereside, Alderley Park, Macclesfield, Cheshire SK10 4TG, UK

### MONOTHERAPY FOR ADVANCED COLORECTAL CANCER

Raltitrexed is now available in more than ten countries for the first-line treatment of advanced colorectal cancer (CRC). Until recently, the only effective chemotherapy for this disease was 5-FU, administered in conjunction with a modulating agent [usually leucovorin (LV)]. Chemotherapy regimens based on 5-FU have prolonged survival significantly compared with best supportive care alone in advanced CRC (Nordic Gastrointestinal Tumour Adjuvant Therapy Group, 1992; Scheithauer et al, 1993; Allen-Mersh et al, 1994), but complex and inconvenient administration schedules are associated with the use of this drug.

The efficacy of raltitrexed as monotherapy in patients with advanced CRC has been shown in three phase III clinical studies (Cunningham et al, 1996*a*; Harper and Study Group, 1997; Kerr, 1997; Pazdur and Vincent, 1997) in which raltitrexed was compared with two standard 5-FU-based regimens. Two international trials, studies 3 (439 patients) and 12 (495 patients), compared raltitrexed 3 mg $m^{-2}$ once every 3 weeks with 5-FU 425 mg $m^{-2}$ plus LV 20 mg $m^{-2}$ (Mayo regimen) and 5-FU 400 mg $m^{-2}$ plus LV 200 mg $m^{-2}$ (Machover regimen), respectively, every 4–5 weeks. A North American trial, study 10, originally randomized patients to three treatment arms: raltitrexed 3 and 4 mg $m^{-2}$ and 5-FU + LV (Mayo regimen). The 4 mg $m^{-2}$ dose of raltitrexed was discontinued because of unacceptable toxicity, and the subsequent analysis was based on a two-group comparison in a total of 427 patients. Objective response rates, times to progression and survival data are shown in Table 1. Palliative benefits of treatment included weight gain and improvements in performance status and disease-related symptoms, and were seen in all trials, with the greatest benefits being seen in patients who achieved complete or partial remission or disease stabilization (45–70% of all patients).

### FUTURE POTENTIAL

Speculation with regard to the potential clinical use of raltitrexed in other tumours, in combination with other cytotoxic agents or as adjuvant therapy in colon cancer, has prompted further evaluation in these settings. Raltitrexed has already been evaluated as monotherapy in the management of other tumour types in phase II trials (Cunningham et al, 1996*b*). Complete and partial responses were seen in patients with breast (26%), ovarian (7%), non-small-cell lung (9%) and pancreatic cancer (12%). Although there were no complete or partial responses to treatment with raltitrexed in a phase II study of 33 patients with hepatocellular carcinoma, there were 'good' minor responses in two patients (8%), one of whom had a marked and sustained decrease (from 188 to 5 $\mu g\ l^{-1}$) in plasma α-fetoprotein levels (Rougier et al, 1997). Three other patients also showed decreases of at least 25% in plasma levels of this tumour marker (range 60–98%).

The concept of combination therapy offers three opportunities for improved efficacy. Firstly, drugs with non-overlapping toxicities that have already shown activity against a particular tumour type may be coadministered. Secondly, agents that act at different phases of the cell cycle may be used, which leads to biochemical synergy. Thirdly, agents that act at the same phase of the cell cycle, but through differing mechanisms, may be used; this leads to an additive effect (Figure 2). For example, raltitrexed and 5-FU both act at S-phase through their inhibition of TS; however, each drug enters the cell and achieves this effect in a different way. These, and other drugs in combination, will be considered in more detail in the following discussion.

### COMBINATION TREATMENTS UNDER INVESTIGATION

The clinical trials currently underway to investigate the efficacy of raltitrexed in a range of different tumour types are shown in Table 2. Treatments to be discussed in detail are as follows: raltitrexed with taxanes (e.g. paclitaxel); raltitrexed with anthracyclines (e.g. doxorubicin); and combinations in colon and rectal cancer that include raltitrexed with platinum compounds (e.g. oxaliplatin, cisplatin); raltitrexed with topoisomerase I inhibitors (e.g. CPT-11); raltitrexed with 5-FU (bolus and infusion); and raltitrexed with radiotherapy.

### Raltitrexed with taxanes

The taxanes, which include paclitaxel and docetaxel, block cell replication at $G_2$/M phase by inhibiting normal spindle formation through the formation of abnormally stable microtubule bundles (Schiff and Horwitz, 1980; Horwitz, 1995). The most impressive clinical activity of paclitaxel has been seen in advanced ovarian and breast cancers (Rowinsky, 1995). Phase II data show similar activity for docetaxel, with both drugs being active in advanced breast cancer refractory to anthracycline therapy (Rowinsky et al, 1990; Rowinsky and Donehower, 1995; Seidman 1995). Anti-tumour activity has also been observed in a range of tumours that are generally refractory to other treatments, including non-small- and small-cell lung, head and neck, oesophageal, bladder and germ cell cancers as well as lymphoma and Kaposi's sarcoma (Rowinsky et al, 1990; Rowinsky and Donehower, 1995).

The taxanes show toxicity profiles that differ from that of raltitrexed, with myelosuppression being their most notable side-effect. Paclitaxel induces neutropenia, hypersensitivity reactions, peripheral neuropathy and cardiac rhythm disturbances (Rowinsky and Donehower, 1995; Ratain, 1997). Docetaxel is associated with fluid retention and skin toxicity and, although nausea, vomiting and diarrhoea may be observed, severe gastrointestinal toxicity is rare (Ratain, 1997). Thus, as well as being of mechanistic interest as candidates for combination with raltitrexed, these agents also have toxicity profiles that largely do not overlap with that of raltitrexed.

As shown in Table 2, combination therapy with raltitrexed and paclitaxel is currently under investigation in a phase I clinical study designed to determine maximum-tolerated dose (MTD) in patients with refractory solid tumours. This single-centre USA study is recruiting patients who have undergone previous standard treatment and includes a follow-up to assess safety [adverse events (WHO criteria) and haematology and biochemistry]. Dose levels of raltitrexed 0.5, 1.0, 1.5, 2.0, 2.5 and 3.0 mg $m^{-2}$ are being administered with paclitaxel at a fixed dosage of 175 mg $m^{-2}$ every 3 weeks (this is the highest recommended dosage of paclitaxel in the USA; further escalation of raltitrexed dose in 0.5 mg $m^{-2}$ increments will be undertaken if the MTD is not reached at 3.0 mg $m^{-2}$). Future development may include combination of the agreed dosage of raltitrexed plus paclitaxel with carboplatin in a follow-on phase I study that could lead to randomized comparisons with standard therapy in patients with non-small-cell lung cancer.

### Raltitrexed with anthracyclines

The anthracyclines, which include doxorubicin and daunorubicin, have been in clinical use since the 1960s and form one of the most commonly used groups of anti-cancer drugs. They have several postulated mechanisms of action, the most important of which is believed to be interference with the function of the enzyme topoisomerase II (Ratain, 1997). A representation of the action of anthracyclines is shown in Figure 3.

**Figure 1** Sites of action of raltitrexed, 5-fluorouracil (5-FU) and methotrexate (MTX). dUMP, deoxyuridine monophosphate; FdUMP, 5-fluorodeoxyuridine monophosphate; TMP, thymidine monophosphate

© Cancer Research Campaign 1998

**Table 1** Clinical efficacy results from studies of raltitrexed in advanced colorectal cancer

| | Study 3 | | Study 10 | | Study 12 | |
|---|---|---|---|---|---|---|
| **Parameter** | **Raltitrexed ($n$ = 223)** | **5-FU + LV ($n$ = 216)** | **Raltitrexed ($n$ = 217)** | **5-FU + LV ($n$ = 210)** | **Raltitrexed ($n$ = 247)** | **5-FU + LV ($n$ = 248)** |
| Complete response rate (%) | 3.6 | 3.7 | 2.8 | 1.4 | 3.2 | 3.6 |
| Partial response rate (%) | 15.7 | 13.0 | 11.5 | 13.8 | 15.4 | 14.5 |
| Time to progression (months) | 4.8 | 3.6 | 3.1 | 5.3*** | 3.9 | 5.1** |
| Survival (months) | 10.1 | 10.2 | 9.7 | 12.7* | 10.7 | 11.8 |

*$P$ = 0.01; **$P$ < 0.005; ***$P$ < 0.0001 between groups.

**Figure 2** The cell cycle and some anti-cancer treatments that act on its various phases. 5-FU, 5-fluorouracil

Dose-limiting toxicity of the anthracyclines manifests chiefly as myelosuppression, with neutrophils being primarily affected. Gastrointestinal toxicity, including mucositis, is also common (Ratain, 1997). It should be noted that mucositis is markedly less severe with raltitrexed than with 5-FU (Cunningham et al, 1996*a*) which may make raltitrexed the more suitable of these two TS inhibitors for such a combination study. Anthracyclines are also associated with acute and chronic cardiotoxicity, notably dose-dependent congestive cardiomyopathy.

A phase I multicentre study of raltitrexed 2.5 mg $m^{-2}$ with doxorubicin every 3 weeks in patients with locally advanced or metastatic gastric cancer is being undertaken by the National Cancer Institute of Canada (NCIC). Up to 40 patients with no previous systemic treatment will be recruited to this study, the aim of which is to evaluate objective tumour response and safety. To date, patients have received either 30 or 40 mg $m^{-2}$ of doxorubicin, with no dose-limiting toxicity (DLT) seen at level 1 (raltitrexed 2.5 mg $m^{-2}$ with doxorubicin 30 g $m^{-2}$). Further dose escalations are planned. Future developments include a study of raltitrexed in combination with the second-generation anthracycline epirubicin.

### Raltitrexed with platinum compounds

Cisplatin (Figure 4) was the first anti-cancer platinum compound to enter clinical trials in the 1970s and remains important in the curative therapy of advanced germ cell tumours. Several thousand platinum complexes have been synthesized over the last 20 years in attempts to develop less toxic, non-cross-resistant or oral analogues, one of which is oxaliplatin (McKeage, 1995).

Platinum-containing compounds act by cross-linking (adduction) with DNA strands (cisplatin reacts readily with the purine N7 position to form a variety of mono- and bifunctional DNA adducts, the majority of which are intrastrand cross-links) (Ratain, 1997). The resulting damage to DNA strands (including local kinking and unwinding) causes inhibition of transcription and replication (Figure 5). Cisplatin is the longest-established drug of this type but is associated with severe toxicity (nausea and vomiting, nephrotoxicity, myelosuppression, neurotoxicity and ototoxicity). Oxaliplatin is a third-generation compound that causes minimal myelosuppression and has not been associated with nephrotoxicity; toxicity manifests mainly as nausea and vomiting, diarrhoea and neurotoxicity (Ratain, 1997). These agents have been combined with 5-FU in pretreated patients with resistant tumours, most notably oxaliplatin in patients with advanced CRC (Levi et al, 1992, 1994, 1995; de Gramont et al, 1994).

Platinum compounds and raltitrexed therefore act by different mechanisms: platinum compounds by damaging DNA and raltitrexed by interfering with DNA synthesis and repair. The different toxicity profiles of these compounds add further support to the rationale for their use in combination. Indeed, results of in vitro cell line work indicate synergy between both oxaliplatin and 5-FU in human colonic (HT-29), ovarian (2008, A2780) and hormone-refractory breast (MDA-MB-231) cancer cells (Kelland et al, 1995; Ackland et al, 1996; Raymond et al, 1996). Simultaneous 72-h exposure of A2780 cells to raltitrexed and cisplatin showed synergism at some concentration ratios and additivity or antagonism at others (Table 3).

A phase I dose escalation study is currently under way in France in patients with advanced cancer refractory to previous therapy. Fifteen patients with a variety of tumour types (mesothelioma, small-cell lung, ovarian, stomach, adrenal and duodenal/jejunum cancer) have been recruited to five dosage levels (all treatments given once every 3 weeks) (Table 4). The study is designed to determine the recommended dosage and side-effect profile (with particular reference to haematological, biochemical and neurological toxicities) for raltitrexed with oxaliplatin. In addition, a phase I study of raltitrexed in combination with cisplatin in patients with non-small-cell lung cancer is being carried out in Germany; three patients have so far been recruited to the first dose level of raltitrexed 2.6 mg $m^{-2}$ with cisplatin 60 mg $m^{-2}$. To date, no DLT has been reported in either study.

**Table 2** Ongoing study programme for raltitrexed

| Drug(s) | Malignancy | Investigator | Country | Phase | Regimen |
|---|---|---|---|---|---|
| Raltitrexed | Head and neck | Zalcberg J, Clarke S | Australia | II | 3 mg $m^{-2}$ every 3 weeks |
| Raltitrexed | Advanced soft tissue sarcoma | EORTC | Europe | II | 3 mg $m^{-2}$ every 3 weeks |
| Raltitrexed | Squamous cell head and neck | Samlowski WE and the South West Oncology Group | USA | II | 3 mg $m^{-2}$ every 3 weeks |
| Raltitrexed | Hormone-resistant prostate | Burch PA and the North Central Cancer Treatment Group | USA | II | 3 mg $m^{-2}$ every 3 weeks |
| Raltitrexed | Advanced paediatric cancers | Adamson PC and the Childrens Cancer Group | USA | I | 2, 2.5, 3, 3.5 or 4 mg $m^{-2}$ every 3 weeks (further escalations allowed if MTD not reached at these dosages) |
| Raltitrexed | Paediatric leukaemia | Weitman SD and the Paediatric Oncology Group | USA | I | 2.5, 3, 3.6 or 4.3 mg $m^{-2}$ every 3 weeks (further escalations allowed if MTD not reached at these dosages) |
| Raltitrexed + paclitaxel | Solid tumours | Vokes EE | USA | I | Raltitrexed 0.5, 1, 1.5, 2, 2.5 and 3.0 mg $m^{-2}$ + paclitaxel 175 mg $m^{-2}$ every 3 weeks. Further raltitrexed dose escalations permitted if MTD not reached |
| Raltitrexed + doxorubicin | Gastric | Seymour L | Canada | I | Raltitrexed 2.5 mg $m^{-2}$ + doxorubicin 30 or 40 mg $m^{-2}$ every 3 weeks |
| Raltitrexed + CPT-11 | Advanced colorectal | Cunningham D | UK | I | Raltitrexed 2, 2.6 or 3 mg $m^{-2}$ + CPT-11 175, 200, 250, 300 or 350 mg $m^{-2}$ every 3 weeks |
| Raltitrexed + oxaliplatin | Small-cell lung, mesothelioma, ovarian, stomach, adrenal, duodenal | Armand JP | France | I | Raltitrexed 2, 2.5 or 3 mg $m^{-2}$ + oxaliplatin 85, 100 or 130 mg $m^{-2}$ every 3 weeks |
| Raltitrexed + cisplatin | Non-small-cell lung | Manegold C | Germany | I | Raltitrexed 2.6 mg $m^{-2}$ + cisplatin 60 mg $m^{-2}$ every 3 weeks |
| Raltitrexed + 5-FU bolus | Advanced colorectal | Schwartz GK | USA | I | Raltitrexed 1.5, 2, 2.5 or 3 mg $m^{-2}$ every 3 weeks + 5-FU to 1500 mg $m^{-2}$ by rapid i.v. infusion 24 h after raltitrexed |
| Raltitrexed + 5-FU infusion | Advanced colorectal | Harstrick A | Germany | I | Raltitrexed 2.6 or 3 mg $m^{-2}$ on weeks 2 and 5 + 5-FU 1200, 1600, 2000 or 2400 mg $m^{-2}$ over 24 h on weeks 1, 2, 3, 4 and 5 |
| Raltitrexed + radiotherapy | Rectal | Price P | UK | I | Raltitrexed 2, 2.6 or 3 mg $m^{-2}$; 2 doses – days 1 and 22 + radiotherapy 28 fractions of 1.8 Gy five times per week for 5–6 weeks |

MTD, maximum-tolerated dose.

### PHASE I STUDIES IN COLON AND RECTAL CANCER

### Raltitrexed with topoisomerase inhibitors

Growing and dividing cells must be able to copy their DNA, either to produce an RNA template for protein synthesis (transcription) or to form more DNA for daughter cells (replication). In both cases, the DNA double helix must be unwound and the strands separated to expose single short sections that act as templates. When this happens, torsional strain is placed on neighbouring sections, and the enzyme topoisomerase I acts to relieve this strain. The enzyme binds covalently to DNA and causes a transient single-strand break that allows the broken ends to rotate and so release the torsional strain. The break is then religated and the process of cell division can continue. Topoisomerase I inhibitors, such as CPT-11 (irinotecan), prevent the religation step and leave the enzyme bound to DNA at a single-strand break (Armand et al, 1995; Verweij et al, 1995). Further DNA unwinding cannot then occur, transcription and replication stop, and cell division ceases (Figure 6).

A number of topoisomerase I inhibitors have been synthesized: these include CPT-11, topotecan, GI 147211, 9-aminocamptothecin and DX 8951. Of these, CPT-11 has shown activity against CRC in phase II trials, with response rates of 15–32% in

© Cancer Research Campaign 1998

**Figure 3** Action of anthracycline drugs on protein synthesis

**Figure 4** Structural formula of cisplatin. Note the square-planar configuration with ammonia and chloride substitutions in the *cis* orientation

**Table 3** Degrees of synergism and additivity with varying concentration ratios of raltitrexed and cisplatin in human ovarian A2780 cancer cells (Kelland et al, 1995)

| | | | | |
|---|---|---|---|---|
| Concentration ratio – raltitrexed:cisplatin | 10:1 | 1:1 | 1:10 | 1:100 |
| Combination index (CI) | 0.81 | 1.6 | 0.98 | 0.84 |

Combination index (CI) is determined from regression analysis of growth inhibition data. CI < 1 indicates synergism; CI = 1 indicates additivity; CI > 1 indicates antagonism.

**Figure 5** Action of platinum compounds on DNA

previously untreated and 5-FU-resistant tumours (Armand et al, 1995; Rougier and Bugat, 1996; Van Cutsem et al, 1996). As both raltitrexed and CPT-11 have demonstrated activity against CRC, and are known to act by different molecular mechanisms, there is a possibility that synergism may be observed if both drugs are given together in patients with this disease.

Preclinical in vitro studies carried out in cloned human cell lines with raltitrexed and SN 38, the active metabolite of CPT-11, have shown evidence of synergism between the two agents (Aschele et al, 1995, 1996*a* and *b*). In particular, Aschele et al (1996*a*) showed that sequential rather than simultaneous administration resulted in synergistic activity, with the magnitude of potentiation being greater when short-term (4-h) exposure was used and SN 38 given first. Higher relative doses of raltitrexed also resulted in increased cytotoxicity (Table 5). These data indicate the importance of optimal scheduling of drug combinations and formed the basis of the schedule selected for the phase I clinical study described later in this section.

Dose-limiting toxicities in phase II trials of CPT-11 have been diarrhoea and neutropenia. Nausea and vomiting also occur frequently. However, mucositis was rare with this drug (Rougier and Bugat, 1996). The major toxicities associated with raltitrexed were elevated transaminases and diarrhoea (Zalcberg, 1997). These toxicity profiles partly overlap, and a phase I dose escalation study is under way to optimize dosages of these drugs when given in combination. This single-centre UK study in 25 patients with advanced metastatic CRC resistant to infusional 5-FU will determine MTD, toxicity and objective response rate. CPT-11 is administered 1 h before raltitrexed, and drugs are administered once every 3 weeks. Dosage levels are shown in Table 6. Partial remission of disease has been reported for a patient on the highest dose level, and there have been no observations of major toxicity, which leads to the interesting speculation that raltitrexed may moderate the toxicity of CPT-11.

### Raltitrexed with 5-FU

As described previously, raltitrexed and 5-FU have both been shown in clinical studies to be effective as single agents in the treatment of advanced CRC. Both drugs share TS as a common target, but raltitrexed inhibits this enzyme directly and specifically. Some metabolites of 5-FU are incorporated into RNA, so 5-FU inhibits both DNA and RNA synthesis. The degree of RNA inhibition appears to be schedule dependent (Sobrero et al, 1997). A combination of the two drugs may therefore produce a more complete blockade of TS than either agent alone. Furthermore, increased activity against heterogeneous cell populations might be conferred by the exclusivity of modes of drug uptake and metabolism. As

**Table 4** Three-weekly dosages of raltitrexed and oxaliplatin in combination in patients with advanced cancer

| Dosage level | –I | I | II | III | IV |
|---|---|---|---|---|---|
| Oxaliplatin (mg m$^{-2}$) | 85 | 85 | 110 | 110 | 130 |
| Raltitrexed (mg m$^{-2}$) | 2.0 | 2.5 | 2.5 | 3.0 | 3.0 |

**Figure 6** The action of topoisomerase I on DNA transcription both drugs are active in advanced CRC, combination of the two would be expected to enhance their respective cytotoxic effects.

Evidence from preclinical studies supports the use of combination therapy with raltitrexed and 5-FU. Raltitrexed is already known to increase the incorporation of fluorouracil phosphate into RNA if administered before 5-FU (Izzo et al, 1995). Preclinical studies with human CRC cell lines HT-29 and HCT-8 have shown synergistic interactions between raltitrexed and 5-FU (Chang et al, 1994; Izzo et al, 1995; Kimbell et al, 1996). When 5-FU was given for 1 h before 24-h incubation with raltitrexed, strong antagonism of the cytotoxic effect was seen with high doses of 5-FU and low doses of raltitrexed. However, low doses of 5-FU and high doses of raltitrexed resulted in synergism. Harstrick (1995) has reported that an overall pattern of additivity is shown by isobole analysis of this sequence (Figure 7). Notably, reversal of the schedules, so that raltitrexed was used first, caused synergism for all drug ratios (Izzo, 1995).

The main toxic effects of 5-FU are mucositis and diarrhoea, myelosuppression and skin disorders. As well as diarrhoea, raltitrexed is associated with leucopenia, and so myelosuppression is common to both agents. However, the severity of this effect may be reduced if 5-FU is given as a prolonged infusion; one of the clinical trials described in this section takes advantage of this. Furthermore, mucositis and leucopenia appear to be markedly less frequent and less severe with raltitrexed than with 5-FU (Zalcberg, 1997).

A review of preclinical and clinical literature on the anti-tumour and toxic effects of 5-FU has indicated that this drug appears to act in different ways when given according to different dose schedules, most notably bolus or continuous infusion (Sobrero, 1997). To investigate this premise, two phase I clinical studies of raltitrexed in combination with 5-FU in patients with advanced CRC are under way. The first, which is being conducted in the USA, involves patients with advanced metastatic or recurrent, unresectable colorectal cancer (Schwartz et al, 1997). Objectives are to determine the MTD, toxicity and anti-tumour activity of raltitrexed followed after 24 h by 5-FU given as a rapid intravenous infusion. Follow-up is 28 days after the final dose and tolerability is to be assessed in terms of haematology, biochemistry and adverse event reports. Dosage levels have been raltitrexed 0.5, 1.0, 1.5, 2.0, 2.5 or 3.0 mg m$^{-2}$ with 5-FU 900 mg m$^{-2}$ every 3 weeks; these have recently been increased to raltitrexed 3.0 mg m$^{-2}$ with 5-FU 1200 mg m$^{-2}$, with the intention to further increase the 5-FU dose to 1500 mg m$^{-2}$. Preliminary data in 12 patients who have received raltitrexed at a dose of up to 2.0 mg m$^{-2}$ showed two partial responses in pretreated patients. In addition, six patients had stable disease lasting from 3.7 to at least

**Table 5** Synergism (indicated by combination indices) between raltitrexed (R) and SN 38, the active metabolite of CPT-11, in HCT-8 human colon cancer cells (Aschele et al, 1995; 1996*a*)

| Inhibition of cell proliferation (%) | Sequential 4-h exposure (dose ratio) | | Sequential 24-h exposure (dose ratio) | |
|---|---|---|---|---|
| | SN38-R (1:10) | R-SN38 (10:1) | SN38-R (1:5) | R-SN38 (5:1) |
| 50 | 0.42 | 0.46 | – | – |
| 75 | 0.11 | 0.27 | – | – |
| 90 | 0.03 | 0.16 | 0.69 | 0.57 |
| 95 | 0.02 | 0.12 | 0.57 | 0.45 |

Combination index (CI) is determined from regression analysis of growth inhibition data. CI < 1 indicates synergism; CI = 1 indicates additivity; CI > 1 indicates antagonism.

**Table 6** Three-weekly dosages of raltitrexed and CPT-11 in combination in patients with advanced colorectal cancer

| Dosage level | –I | I | II | III | IV | V | VI |
|---|---|---|---|---|---|---|---|
| CPT-11 (mg m$^{-2}$) | 175 | 175 | 200 | 250 | 250 | 300 | 350 |
| Raltitrexed (mg m$^{-2}$) | 2.0 | 2.6 | 2.6 | 2.6 | 3.0 | 3.0 | 3.0 |

© Cancer Research Campaign 1998

**Figure 7** Isobole analyses of sequential exposure of HCT-8 and HT-29 human colorectal cancer cells to 5-fluorouracil (5-FU) and raltitrexed. (**A**) Raltitrexed (24 h) followed by 5-FU (1 h); pattern suggests synergism. (**B**) 5-FU (1 h) followed by raltitrexed (24 h); pattern suggests additivity

7.5 months (Schwartz et al, 1997). Most recent data from 17 patients indicate that 70% have stable disease (some for up to 12 cycles) and show a complete response in a non-pretreated patient. There have been some dose delays due to neutropenia but none of the affected patients have withdrawn from treatment. No DLT or grade 3–4 toxicity has been reported to date.

The second phase I study is being conducted at a single German centre in patients with untreated advanced or metastatic CRC. Objectives are to determine the MTD and to assess the pharmacokinetic and pharmacodynamic profile of raltitrexed on days 8 and 29 with 5-FU given by 24-h infusion on days 1, 8, 15, 22 and 29 of each cycle, repeated every 4–5 weeks. Dosage levels are shown in Table 7. To date, level 3 has been reached in ten patients with no DLT and one partial response (at level 2).

**Table 8** Enhancement of cytotoxicity of radiation by raltitrexed or 5-FU in human HT-29 colon cancer cells (Moertel, 1994)

| | Enhancement ratios[a] | | | |
|---|---|---|---|---|
| | Euoxic | | Hypoxic | |
| Treatment | 2.5 Gy | 5 Gy | 2.5 Gy | 5 Gy |
| Raltitrexed 1 μM | 7.7 | 55.7 | 5.5 | 8.6 |
| Raltitrexed 0.5 μM | 7.2 | 35.5 | 4.8 | 6.4 |
| 5-FU 10 μM | 3.9 | 6.1 | 5.9 | 7.1 |

[a]Enhancement ratios were calculated from ratios of surviving fractions of cells at 2.5 Gy and 5 Gy with and without adjuvant drug treatment. Values > 1 indicate enhanced cell killing compared with radiation alone.

### Raltitrexed with radiotherapy

If detected early, colon cancer may be cured by surgery; cure rates of 90% have been reported for Dukes' stage A or B1 (Moertel, 1994). However, rectal cancer is not as readily cured in this way because the close confines of the pelvic bones prevent access by the surgeon to an adequate tumour-free margin. Because of this, local recurrence of rectal cancer is common.

Radiotherapy is often prescribed in an attempt to reduce the risk of tumour recurrence. Administration of post-operative 5-FU in combination with radiotherapy resulted in a 15% survival advantage and lower incidences of metastases and local recurrence in one study (Mayer et al, 1989). These promising results with a TS inhibitor, coupled with the known efficacy of raltitrexed in advanced disease, make the use of raltitrexed as adjuvant therapy with radiotherapy in colorectal cancer an attractive treatment that merits further investigation. Preclinical data, obtained in human HT-29 cancer cells, showed that raltitrexed and 5-FU both enhance cell killing by radiation. However, raltitrexed was a more effective addition than 5-FU (Teicher and Coleman, 1997). Cells were tested under euoxic and hypoxic conditions. As shown in Table 8, raltitrexed 0.5 or 1 μM increased radiation-induced cell killing to a markedly greater extent than 5-FU 10 μM under euoxic conditions. In the transplantable Lewis lung carcinoma cell line, raltitrexed showed a radiation dose-modifying effect of 1.5. Raltitrexed is believed to achieve this effect through the inhibition of repair of radiation-induced DNA strand breaks in euoxic and hypoxic cells and by interaction with radiation in hypoxic cells.

Two phase I studies in the UK are currently investigating combinations of raltitrexed 2.0, 2.6 and 3.0 mg $m^{-2}$ with radiotherapy (given as 28 fractions of 1.8 Gy 5 times weekly for 5–6 weeks) in patients with resected or inoperable/recurrent rectal

**Table 7** Doses of raltitrexed (days 8 and 29) and 5-FU infusion (days 1, 8, 15, 22 and 29) in patients with advanced colorectal cancer

| Dosage level (4- to 5-weekly cycles) | 1 | 2 | 3 | 4 | 5 | 6 |
|---|---|---|---|---|---|---|
| Raltitrexed 15-min infusion (mg $m^{-2}$) | 2.6 | 2.6 | 2.6 | 2.6 | 3.0 | 3.0 |
| 5-FU 24-h infusion (mg $m^{-2}$) | 1200 | 1600 | 2000 | 2400 | 2000 | 2400 |

© Cancer Research Campaign 1998

**Figure 8** Dosing scheme for raltitrexed with radiotherapy in patients with resected rectal cancer cancer (Figure 8). The investigators aim to recruit 18 patients to each study to determine the optimum dosage of raltitrexed to be used as combination treatment with radiotherapy. As in other studies discussed, safety is being assessed in terms of adverse event reports and haematological and biochemical parameters. MTD has not yet been reached at level 2, and the major toxicities to date are asthenia and diarrhoea. Interestingly, raltitrexed does not appear to sensitize normal tissue to radiation.

### CONCLUSIONS

Following the extensive monotherapy clinical trial programme in advanced CRC, raltitrexed is now being investigated in combination with other cytotoxic agents and as adjuvant treatment for earlier stages of the disease. The direct and specific mode of action of raltitrexed offers exciting new opportunities for more effective cytotoxic treatments in a range of malignancies. Initial encouraging results in other tumour types have led to the initiation of monotherapy and combination studies in head and neck, prostate, lung, breast, gastric, colorectal, ovarian and adrenal cancers, and in paediatric malignancies and advanced soft tissue sarcoma. The significant single-agent anti-tumour activity (comparable to modulated 5-FU regimens in advanced CRC) and early evidence of synergistic activity in combination with 5-FU and/or CPT-11 offer for the first time the possibility of a stepped increase in the effectiveness of chemotherapy in this disease. The lack of toxicity seen with raltitrexed in combination with CPT-11 is both scientifically interesting and clinically exciting, and indicates that improvements in efficacy may be achieved with no deterioration in tolerability.

Available data from the combination studies show that all treatments have been well tolerated so far. As further data emerge, it will be possible to evaluate more fully the broad contribution of raltitrexed to the treatment of cancer.

### REFERENCES

Ackland S, Kuiper CM, Garg M, Bergman AM, Smid K and Peters GJ (1996) Variable effects of the combination of Tomudex (ZD1694) and cisplatin in ovarian cancer cell lines (abstract). *Ann Oncol* **7**: 7

Allen-Mersh TG, Earlam S, Fordy C, Abrams K and Houghton J (1994) Quality of life and survival with continuous hepatic-artery floxuridine infusion for colorectal liver metastases. *Lancet* **344**: 1255–1260

Armand J, Ducreux M, Mahjoubi M, Abigerges D, Bugat R, Chabot G, Herait P, de Forni M and Rougier P (1995) CPT 11 (Irinotecan) in the treatment of colorectal cancer. *Eur J Cancer* **31A**: 1283–1287

Aschele C, Baldo C, Guglielmi A and Sobrero A (1995) In vitro synergism between Tomudex and SN-38 in human colon cancer cells (abstract 400). *Tumori* **81** (suppl.): 132

Aschele C, Baldo C, Guglielmi A, Sobrero A and Bertino JR (1996*a*) Sequence dependent synergism between Tomudex and Irinotecan in human cancer cells *in vitro* (abstract 302). *Ann Oncol* **7** (suppl. 1): 88

Aschele C, Sobrero A, Baldo C, Ardizzoni A, Bornmann WG and Bertino JR (1996*b*) In vitro synergism between SN-38 and Tomudex (TX): importance of scheduling and dose ratio (abstract 621P). *Ann Oncol* **7**(suppl. 5): 129

Chang YM, Zielinski Z and Izzo J (1994) Pretreatment of colon carcinoma cells to D1696 (Tomudex) markedly enhances 5-fluorouracil toxicity (abstract). *Proc Am Assoc Cancer Res* **35**: A1966

Cunningham D, Zalcberg JR, Rath U, Oliver I, van Cutsem E, Svensson C, Seitz JF, Harper P, Kerr D, Perez-Manga G and the Tomudex Colorectal Cancer Study Group (1996*a*) Final results of a randomised trial comparing 'Tomudex'® (raltitrexed) with 5-fluorouracil plus leucovorin in advanced colorectal cancer. *Ann Oncol* **7**: 961–965

Cunningham D, Zalcberg J, Smith I, Gore M, Pazdur R, Burris H IIIrd, Meropol NJ, Kennealey G and Seymour L (1996*b*) 'Tomudex' (ZD1694): a novel thymidylate synthase inhibitor with clinical antitumour activity in a range of solid tumours. *Ann Oncol* **7**: 179–182

de Gramont A, Gastiaburu J, Tournigand C, Louvet C, Varette C, Raymond E, Lecouturier S, Brienza S and Krulik M (1994) Oxaliplatin with high-dose folinic acid and 5-fluorouracil 48 h infusion in pretreated metastatic colorectal cancer (abstract). *Proc Am Soc Clin Oncol* **13**: 220

Harper P (1997) Advanced colorectal cancer (ACC): results from the latest (raltitrexed) Tomudex comparative study (abstract 802). *Proc Am Soc Clin Oncol* **16**: 228a

Harstrick A, Schleucher N, Gonzales A, Schmidt C, Hoffmann A, Wilke H, Rustum Y and Seeber S (1995) Interactions and cross resistance patterns between various schedules of 5-FU and the new, folate-based thymidylate synthase inhibitor 'Tomudex' (D1694). *Eur J Cancer* **31A**(suppl. 5): S30

Horwitz SB (1994) Taxol (paclitaxel): mechanisms of action. *Ann Oncol* **5**: S3–S6

Izzo J, Zielinski Z, Chang YM and Bertino JR (1995) Molecular mechanisms of the synergistic sequential administration of D1694 (Tomudex) followed by FUra in colon carcinoma cells (abstract). *Proc Am Assoc Cancer Res* **36**: 381

Jackman AL and Gibson W (1995) Polyglutamation of the thymidylate synthase inhibitor, ZD 1694 (Tomudex) in normal mouse tissues (abstract). *Proc Am Assoc Cancer Res*, **36**: 377

Jackman AL and Judson IR (1996) The new generation of thymidylate synthase inhibitors in clinical study. *Exp Opin Invest Drugs* **5**: 719–736

Jackman AL, Taylor GA, Gibson W, Kimbell R, Brown M, Calvert AH, Judson IR and Hughes LR (1991) ICI D1694, a quinazoline antifolate thymidylate synthase inhibitor that is a potent inhibitor of L1210 tumor cell growth *in vitro* and *in vivo*: a new agent for clinical study. *Cancer Res* **51**: 5579–5586

Jackman AL, Farrugia DC, Gibson W, Kimbell R, Harrap KR, Stephens TC, Azab M and Boyle FT (1995) ZD1694 (Tomudex): a new thymidylate synthase inhibitor with activity in colorectal cancer. *Eur J Cancer* **31A**: 1277–1282

Kelland LR, Kimbell R, Hardcastle A, Aherne GW and Jackman AL (1995) Relationships between resistance to cisplatin and antifolates in sensitive and resistant tumour cell lines. *Eur J Cancer* **31A**: 981–986

Kerr DJ (1997) Clinical efficacy of 'Tomudex' (raltitrexed) in advanced colorectal cancer. *Anticancer Drugs* **8** (suppl. 2): S11–S15

 © Cancer Research Campaign 1998

Kimbell R, Brunton L and Jackman AL (1996) Combination studies with Tomudex and 5-fluorouracil in human colon cancer cell lines (abstract). *Br J Cancer* **73**: 29

Levi FA, Misset JL, Brienza S, Adam R, Metzger G, Itzakhi M, Caussanel JP, Kunstlinger F, Lecouturier S and Descorps-Declére A (1992) A chronopharmacologic phase II clinical trial with 5-fluorouracil, folinic acid and oxaliplatin using an ambulatory multichannel programmable pump. *Cancer* **69**: 893–900

Levi FA, Zidani R, Vannetzel J-M, Perpoint B, Focan C, Faggiuolo R, Chollet P, Garufi C, Itzhaki M and Dogliotti L (1994) Chronomodulated versus fixed-infusion-rate delivery of ambulatory chemotherapy with oxaliplatin, fluorouracil, and folinic acid (leucovorin) in patients with colorectal cancer metastases: a randomized multi-institutional trial. *J Natl Cancer Inst* **86**: 1608–1617

Levi FA, Giacchetti S, Adam R, Zidani R, Metzger G and Misset JL (1995) Chronomodulation of chemotherapy against metastatic colorectal cancer. *Eur J Cancer* **31A**: 1264–1270

Mayer RJ, O'Connell MJ, Tepper JE and Wolmark N (1989) Status of adjuvant therapy for colorectal cancer. *J Natl Cancer Inst* **81**: 1359–1364

McKeage MJ (1995) Comparative adverse effect profiles of platinum drugs. *Drug Safety* **13**: 228–244

Moertel CG (1994) Chemotherapy for colorectal cancer. *N Engl J Med* **330**: 1136–1142

Nordic Gastrointestinal Tumour Adjuvant Therapy Group (1992) Expectancy or primary chemotherapy in patients with advanced asymptomatic colorectal cancer. *J Clin Oncol* **10**: 904–911

Pazdur R and Vincent M (1997) Raltitrexed (Tomudex) vs 5-fluorouracil + leucovorin (5-FU + LV) in patients with advanced colorectal cancer (ACC): results of a randomised multicenter North American trial (abstract). *Proc Am Soc Clin Oncol* **16**: 228a

Ratain MJ (1997) Pharmacology of cancer chemotherapy. In *Cancer: Principles and Practice of Oncology*, De Vita Jr VT, Hellman S and Rosenberg SA. (eds), pp. 375–512. Lippincott-Raven: Philadelphia, PA

Raymond E, Djelloul S, Buquet Fagot C, Goldwasser F, Mester J, Cvitkovic E, Louvet C and Gespach C (1996) Oxaliplatin (LOHP) and cisplatin (CDDP) in combination with 5FU, specific thymidylate synthase (TS) inhibitors (AG337, ZD1694), and topoisomerase I (Topo-I) inhibitors (SN38, CPT-11) in human colonic, ovarian and breast cancers (abstract). *Proc Am Assoc Cancer Res* **37**: 291

Rougier PH and Bugat R (1996) CPT-11 in the treatment of a colorectal cancer: clinical efficacy and safety profile. *Semin Oncol* **23**: 34–41

Rougier PH, Ducreux M, Kerr D, Carr BI, François E, Adenis A and Seymour A (1997) A phase II study of raltitrexed ('Tomudex') in patients with hepatocellular carcinoma. *Ann Oncol* **8**: 500–502

Rowinsky EK and Donehower RC (1995) Drug therapy: paclitaxel (Taxol). *N Engl J Med* **332**: 1004–1014

Rowinsky EK, Cazenave LA and Donehower RC (1990) Taxol: a novel investigational antineoplastic agent. *J Natl Cancer Inst* **82**: 1247

Rustum YM, Harstrick A, Cao S, Vanhoefer U, Yin M-B, Wilke H and Weeber S (1997) Thymidylate synthase inhibitors in cancer therapy: direct and indirect inhibitors. *J Clin Oncol* **15**: 389–400

Scheithauer W, Rosen H, Kornek G-V, Sebesta C and Depisch D (1993) Randomised comparison of combination chemotherapy plus supportive care with supportive care alone in patients with metastatic colorectal cancer. *Br Med J* **306**: 752–755

Schiff PB and Horwitz SB (1980) Taxol stabilizes microtubules in mouse fibroblast cells. *Proc Natl Acad Sci USA* **77**: 1561–1565

Schwartz GK, Kemeny N, Saltz L, Sugarman A, Danso D, Kelsen DK, Tong W and Bertino J (1997) Phase I trial of sequential Tomudex® (TOM) and 5-fluorouracil (5-FU) in patients with advanced colorectal cancer (abstract 728). *Proc Am Soc Clin Oncol* **16**: 208a

Seidman AD (1995) The emerging role of paclitaxel in breast cancer therapy. *Clin Cancer Res* **1**: 247

Sobrero AF, Aschele C and Bertino JR (1997) Fluorouracil in colorectal cancer – a tale of two drugs: implications for biochemical modulation. *J Clin Oncol* **15**: 368–381

Teicher BA and Coleman CN (1997) Enhanced radiation response by 'Tomudex' *in vitro* and *in vivo* (abstract). *Proc Am Assoc Cancer Res* **38**: 537–538

Touroutoglou N and Pazdur R (1996) Thymidylate synthase inhibitors. *Clin Cancer Res* **2**: 227–243

Van Cutsem E, Cunningham D, Ten Bokkel Huinink W, Dirix L, Punt C, Alexopoulos C, Cote C, Blanc C and Bleiberg H (1996) Irinotecan (CPT-11) multicenter phase II study in colorectal cancer patients with documented progressive disease on prior 5FU: preliminary results. *Proc Am Soc Clin Oncol* **15**: 562

Verweij J and Schellens J (1995) Topoisomerase I inhibition: a new target or new missiles? *Ann Oncol* **6**: 102–104

Zalcberg JR (1997) Overview of the tolerability of 'Tomudex' (raltitrexed): collective clinical experience in advanced colorectal cancer. *Anticancer Drugs* **8**: S17–S22

© Cancer Research Campaign 1998